# A crack templated copper network film as a transparent conductive film and its application in organic light-emitting diode

**DOI:** 10.1038/s41598-022-24672-x

**Published:** 2022-11-28

**Authors:** Ping Liu, Bing Huang, Lei Peng, Liming Liu, Qingguo Gao, Yuehui Wang

**Affiliations:** 1 College of Electron and Information Engineering, University of Electronic Science and Technology of China Zhongshan Institute, Zhongshan, 528402 China; 2 School of Physics, University of Electronic Science and Technology of China, Chengdu, 610054 China

**Keywords:** Materials for devices, Nanoscale materials

## Abstract

In this paper, a highly transparent, low sheet resistance copper network film fabricated by a crack template, which made by drying an acrylic based colloidal dispersion. The fabricated copper network film shows excellent optoelectronic performances with low sheet resistance of 13.4 Ω/sq and high optical transmittance of 93% [excluding Polyethylene terephthalate (PET) substrate] at 550 nm. What’s more, the surface root mean square of the copper network film is about 4 nm, and the figure of merit is about 380. It’s comparable to that of conventional indium tin oxide thin film. The repeated bending cycle test and adhesive test results confirm the reliability of the copper network film. As a transparent conductive film, the copper network film was used as an anode to prepare organic light-emitting diode (OLED). The experiment results show that the threshold voltage of the OLED is less than 5 V and the maximum luminance is 1587 cd/m^2^.

## Introduction

Organic light-emitting diode (OLED) has attracted more and more attention, due to the characteristics of spontaneous light, ultra-thin, high contrast, low power consumption and simple structure. One of the important components in OLED is transparent conductive film (TCF). Indium tin oxide (ITO) thin films are usually used as the anodes for Organic light-emitting diodes (OLEDs). The ITO thin films don’t meet the requirement of flexible equipment due to the characteristic of fragility, high cost and complex preparation process. In addition, The ITO thin films are easy to crack under bending, which leads to degradation of device performance^1^. Besides, The ITO thin films have significant light reflection characteristics^2^. Bending and impact resistance are the basic requirements of flexible equipment. Although there are some ITO films prepared on Polyethylene terephthalate (PET) substrate that can be used for flexible optoelectronic devices, their performances are lower than those ITO films prepared on some rigid substrates. Some researchers have started looking for alternatives of ITO, such as metal thin films. Recently, various substitutes have been developed. These transparent conductive films (TCFs) can be mainly divide into three categories. Carbon-based TCFs (graphene^3^, carbon nanotubes (CNTs)^4^); metal based TCFs (metallic nanowires^5,6^ and metallic network^7–9^) and hybrid TCFs^10–12^.

Complete and defect-free graphene has high carrier mobility and high transmittance. However, the interlayer contact resistance is larger than ITO, and if you want to produce defect-free graphene, the high cost of preparation has to be considered ^13^. CNTs have excellent photoelectric properties, thermal properties and mechanical stability. Carbon nanotube (CNT) was first discovered by Iijima^14^ in 1991. However, it’s difficult to control the length of CNTs and uniformity of diameter. Metallic nanowires can be easily synthesized by solution methods, such as hydrothermal method and polyol method, which is low cost and high efficiency^15^. Metallic nanowires, especially silver nanowires, have good mechanical flexibility and better trade-off between transmittance and electrical conductivity. Amit Kumar and co-workers systematically elaborate an abundant of approaches to forming silver nanowire for fabricating transparent conducting electrodes^16^. A host of metal-based TCFs have been applied in solar cells , biological devices and flexible touchscreen panels and displays^9,12^. It has been proved as an alternative to ITO^17,18^ Hybrid TCFs of Carbon-based materials, metals and conductive polymers can effectively utilize the advantage of a single material and complement the shortcomings of a single material^19,20^. Cu-transparent conductive oxides show a broad work function and work as anode or cathode electrodes in organic light-emitting diodes (LEDs) without hole injection layer^21^. Meanwhile, hybrid transparent electrodes can improve charge injection and obtain better current density^22^. Thus, the hybrid TCFs with suitable energy-level alignments can improve the photoelectric performance and thermochemical stability^23^. As metallic network film, the trade-off between transmittance and conductivity is also better than ITO, what’s more, it has better mechanical flexibility than ITO. Compared with metallic nanowires, there is no need to consider the influence of lap resistance. In addition, the geometry of metallic network film can be manipulated to submicron scale. Which is invisible to human eyes. This characteristic is vital in transparent electronic devices. At the same time, TCFs prepared by metallic network film is superior than ITO has been proved through theory. Yi Cui of Stanford University had testified that the transmittance and sheet resistance are better than ITO by electromagnetic simulation^24^.

The fabrication of metallic network TCFs are as follows, electrospinning^25^, photon sintering ^26–28^ lithography^29–31^, additive manufacturing^32–34^, ink-jet printing^35^, etc. Typically, the preparation of metallic network film is divided into three processes, patterning, metallization and transfer. Patterning means the production of microstructures or nanostructures. The most common patterning methods are photolithography and nanoimprint lithography. During photolithography, rotating a thin layer photoresist on the substrate, then, a mask with the pattern coated under UV light to cover the photoresist. The photoresist is chemically changed when exposed to UV light and a template with micro-nanostructure is obtained after developed.

Crack template is a new method for making transparent conductive metallic network film^36–42^. Compared with nanoimprint lithography or photolithography, the raw materials for Crack template are low cost, usually for acrylic acid or titanium dioxide. Moreover, the making process of crack template is simple, material resources are extensive. Through the crack template method, the metallic network TCFs can be fabricated in large area. Nevertheless, copper has low resistivity and abundant reserves. Compare with silver, there are 1000 times more copper than silver and the price of copper is much cheaper than sliver, almost 1/100 times, but the conductivity is only 6% lower than silver^43^. So, copper network films based on crack templates are expected to be used in low-cost organic devices. One of the basic challenges for preparing copper network films is to obtain high light transmittance maintaining low sheet resistance and low surface roughness.

We have explored the viability of cracks as a template for the preparation of metallic network film from acrylic acid. The fabricated copper network films show excellent optoelectronic performances with low sheet resistance of 13.4 Ω/sq and high optical transmittance of 93% (excluding PET substrate) at 550 nm. The copper network film was used as an anode of a flexible OLED. The threshold voltage of the OLED is less than 5 V and the maximum luminance is 1587 cd/m^2^.

## Experimental section

### Preparation of copper network film

**Figure 1:**
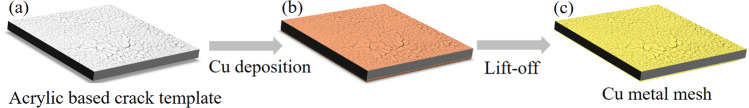
Schematic illustration for preparing copper network films. (**a**) acrylic based crack template; (**b**) deposition of copper on a crack template; (**c**) lift-off the template.

**Figure 2:**
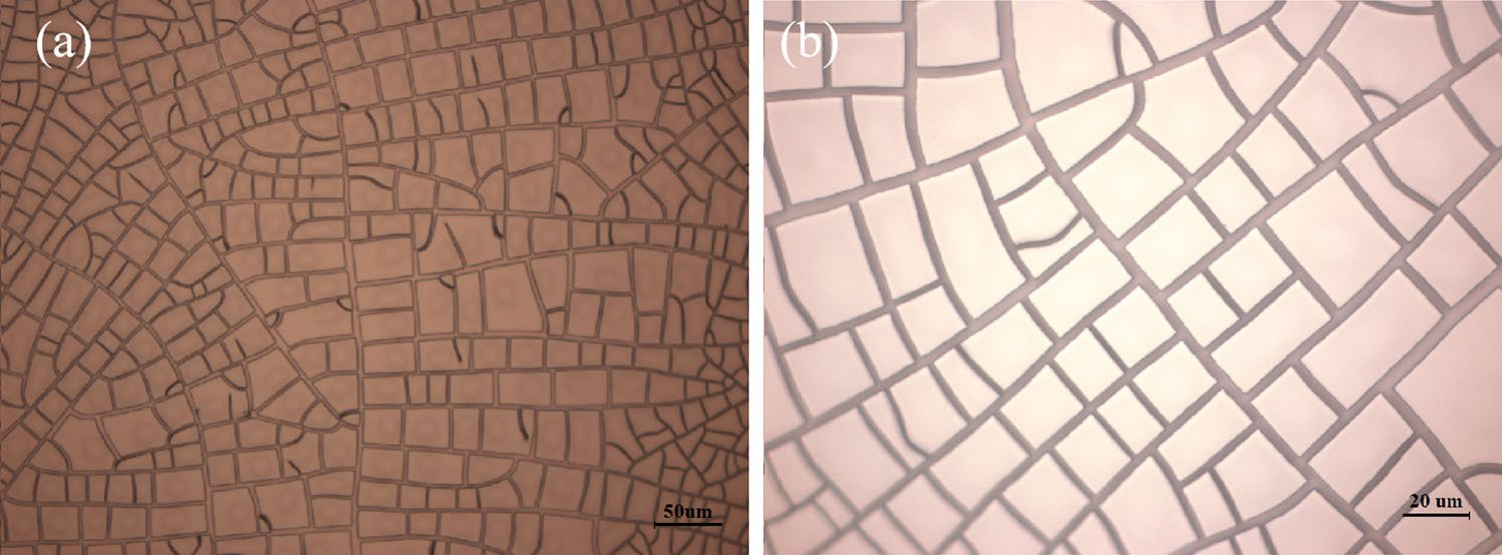
Optical microscope images of crack morphology in different magnification: (**a**) 200 times, (**b**) 500 times.

PET was treated by oxygen plasma, which can improve the hydrophilicity of PET. The higher the hydrophilicity of the substrate, the more conducive to the formation of micro-cracks. The acrylic was diluted with alcohol in a 3:2 volume ratio, and then filter twice through a vacuum filter. The process for preparation copper network films is shown schematically in Fig. 1. Firstly, a crack template was made by drying an acrylic based colloidal dispersion, which coated on PET. After cracking, these cracks are completely detached from the substrate. And then, basing on the template, the copper is deposited by vacuum evaporation. In the final step of lift-off, we use a syringe to drain tetrahydrofuran to flush the surface of the template. Propylene glycol methyl ether acetate (PGMEA) was used for ultrasound for 15 min. As shown in Fig. 2, highly interconnected cracks are obtained spontaneously.

### Fabrication of flexible OLED based on copper network film

**Figure 3:**
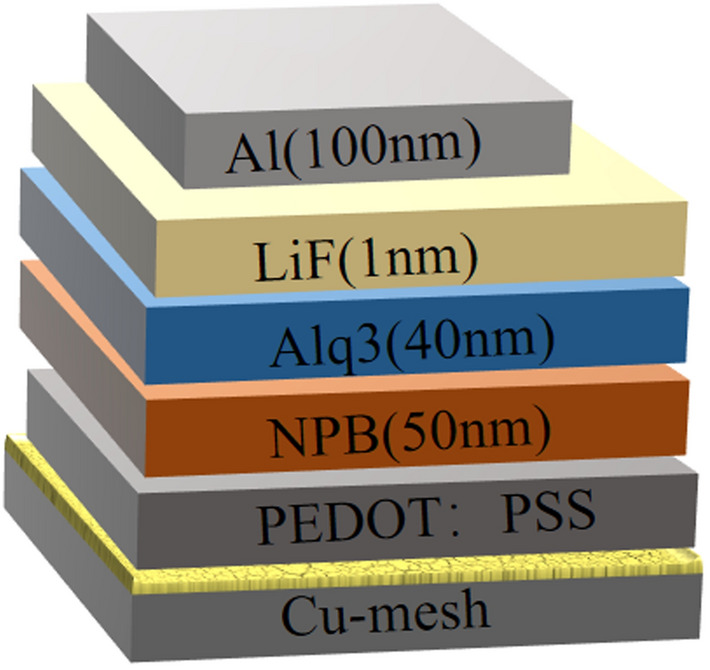
The device structure of the OLED.

In this paper, we have prepared an OLED with a common structure by vacuum evaporation equipment. The structure of OLED as shown in Fig. 3. For OLED, the roughness of TCFs has a significant influence, excessive roughness may cause a short circuit. Therefore, copper network film fabricated at 2000 r/min was used as an anode, the surface root-mean-square (RMS) roughness is about 4 nm. PEDOT:PSS was used as the hole injection layer, *N*,*N*′-bis(1-naphthyl)-*N*,*N*′-diphenyl-1,1′-biphenyl-4,4′-diamine (NPB) was used as the hole transport layer, tris (8-hydroxyquinoline) aluminium (Alq_3_) was used as the electron transport layer and the emitting layer, LiF was used as the electron injection layer, Al was used as the cathode. The device structure of the OLED and thickness of each layer is indicated in Fig. 3.

## Result and discussion

Transmittance was measured with an ultraviolet–visible–near infrared photometer (UH4150, HITACHI) with an integrating sphere attached. Sheet resistance was measured using a four-point probe system (DMR-1C) and multimeter. Optical microscopy images were taken using Olympus BX51M. The characterization of copper network film was measured by AFM (BRUKER, Dimension Edge). Bending test was done using an ordinary pen, which diameter is 9 mm. The adhesion test was performed using a 3 M scotch tape to attach onto the copper network film and then peeled off, records the resistance every 10 times.

### Microstructure of copper network film

**Figure 4:**
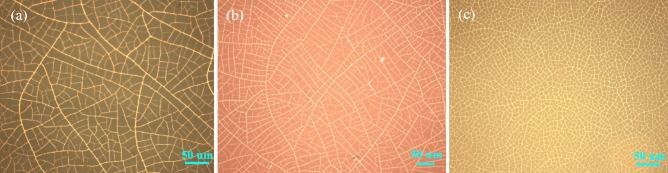
Optical microscope images of copper network films with different coating speeds: (**a**) 1500 r/min, (**b**) 2000 r/min, (**c**) 2400 r/min.

Copper network films fabricated by crack template are shown in Fig. 4, where the inside scale is 50 μm. The density of copper network film is increasing with the increase of coating speed. However, when the coating speed exceeds a certain value, the copper wires started to crack slightly, which affects conductivity (see Fig. 4c). The template was made of acrylic resin, which is rotated for 60 s by a spin coater and then dried at 100 °C on a constant temperature heating table for 1 h.

### Sheet resistance and transmittance of copper network film

**Figure 5:**
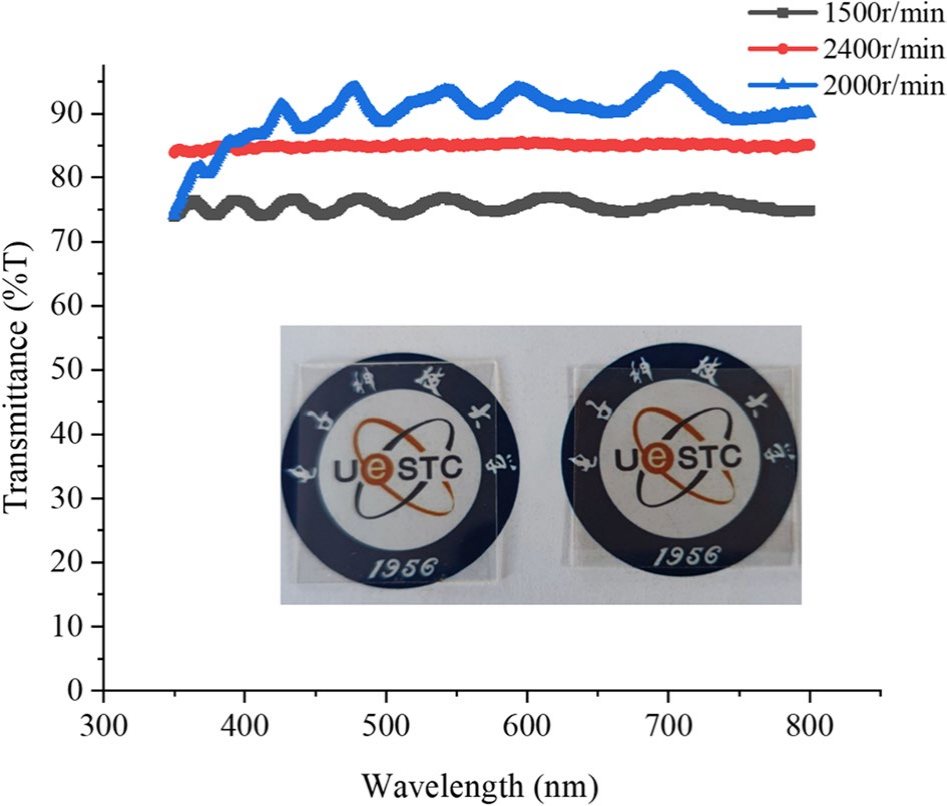
The transmittance of copper network films with different coating speed.

The spin coating speed affects the morphology of the crack template. If the speed is too slow, the width of crack will be too wide. If the speed is too fast, the interconnection of crack is very poor. The wider the crack, the more copper is deposited, which means that copper wires occupy more area, thus affecting the transmittance of the film. When the interconnection of crack is very poor, the conductivity of the film will be affected. Therefore, we chose an appropriate interval, in a valid speed range, as coating speed increases, the thickness of the template decreases and the width of the cracks becomes finer. The transmittance of our copper network TCFs are shown in Fig. 5. These templates were made by three different coating speed. The transmittance of a copper network TCF was 76.5% at 550 nm, whose template was coated at 1500 r/min for 60 s. The sheet resistance of the copper network TCF is 3.4 Ω/sq and the surface RMS roughness is 33.2 nm. It’s worth noting that the transmittance of all copper network films in this paper refers to the transmittance excluding PET substrate. Based on the template fabricated at 2000 r/min, the copper network TCF with transmittance of 93% and sheet resistance of 13.4 Ω/sq were obtained. The RMS roughness of the copper network TCF is as low as 4 nm. The built-in figure is a comparison of transmittance between a clean PET substrate (see the left figure) with a copper network TCF with transmittance of 93% (see the right figure). As you can see that the word UESTC under the TCFs is still can be seen clearly. Based on the template fabricated at 2400 r/min, the transmittance of the copper network TCF is 85.2% and sheet resistance is 7.2 Ω/sq.

**Figure 6:**
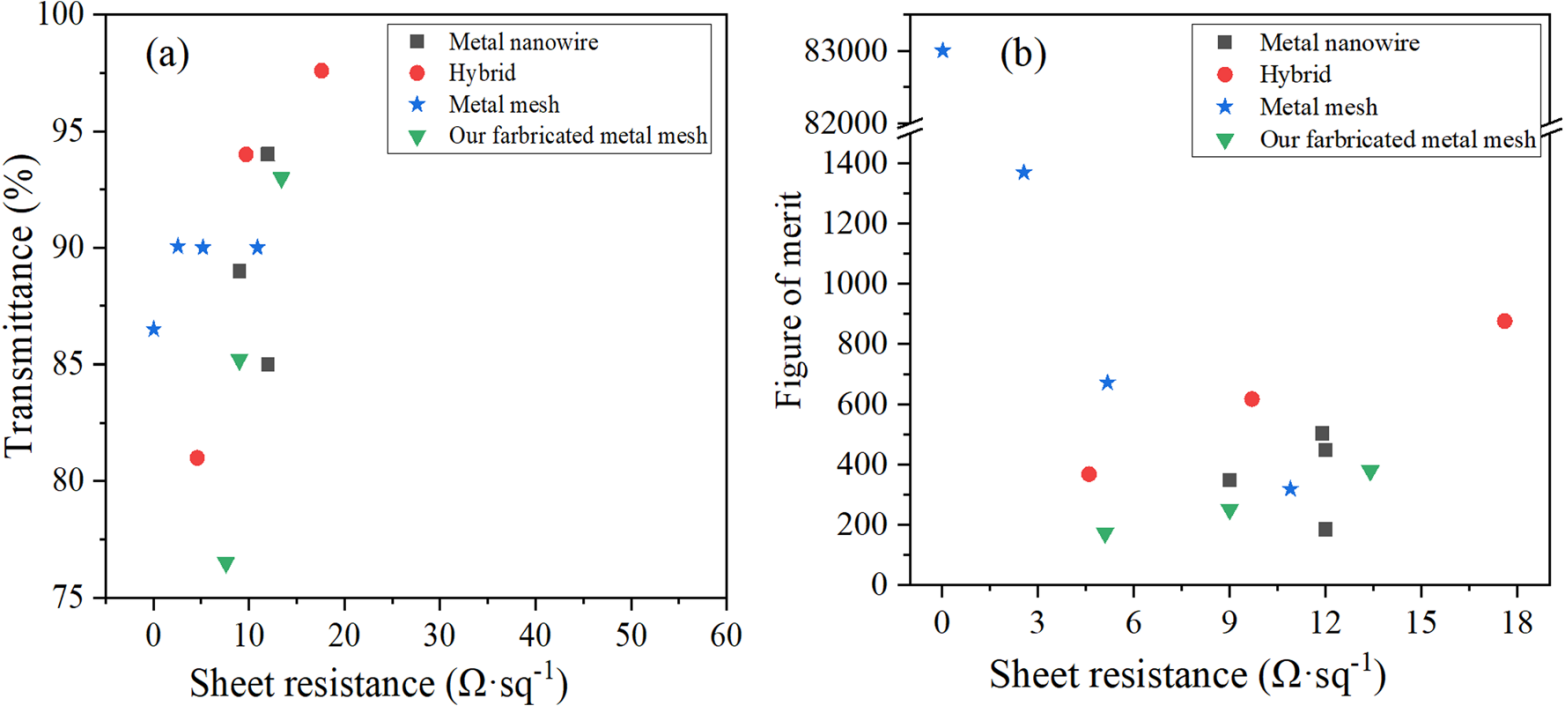
Performances of the flexible TCFs. (**a**) Transmittance versus sheet resistance data of the metallic network in comparison with previous studies (metallic wire^13,44–46^, metallic network^9,25,33,47^, hybrid ^48–50^), (**b**) Comparison of the figure of merit values.

Figure 6 shows the performances comparation of the flexible copper network TCFs. Transmittance at the wavelength of 550 nm and the sheet resistance values are plotted, in order to compare with the result of previous studies (metallic nanowire^13,44–46^ metallic network^9,25,33,47^, hybrid^48–50^). Figure of merit (FoM) were calculated, the calculation formula is as follows^51^

1 $${\text{FoM}} = \frac{{\updelta _{{{\text{dc}}}} }}{{\delta _{{{\text{opt}}}} }} =\frac{{188.5}}{{{\text{R}}_{{\text{s}}} \left( {\frac{1}{{\sqrt {\text{T}} }}-{\text{ 1}}} \right)}},$$

where $\updelta _{{dc}}$ is the electrical conductance and $\updelta _{{opt}}$ is the optical conductance^51^, R_s_ means sheet resistance and T denotes transmittance. As shown in Fig. 6a, the sheet resistance of the TCFs prepared by us is well, while the transmittance of TCFs changes greatly, which is still related to the cleaning operation.

Hybrid type and metallic network type TCFs demonstrated outstanding performance in comparison with metallic nanowire based TCFs. As shown in Fig. 6b, the FoM of our TCFs is about 380, compared with commercial ITO which FoM is about 200, it has certain application prospect.

### Mechanical properties of copper network film

For bulk materials, $\uprho$ is usually used to measure the conductivity of the material. The measurement of conductivity involves the thickness $t$ of the material, but the thickness of TCFs is usually nonuniform, the concept of square resistance is introduced to measure the conductivity of TCFs. As shows in the following formula.

2 $${\text{R}}_{{\text{s}}} = \frac{\uprho }{{\text{t}}},$$

According to Formula (2), $\uprho$ is an intrinsic property of a material, the sheet resistance decreases with the increase of thickness, and the increase of the thickness will bring the decrease of the transmittance. This explains why there is a trade-off between transmittance and conductivity. Due to the use of four-probe test will cause irreversible defects on the film surface, which will seriously affect the subsequent preparation of OLED devices. The sheet resistance is determined by

3 $$\text{R} = {\text{R}}_{\text{s}}\frac{\text{L}}{{\text{W}}},$$

where R is the resistance measured by a multimeter, ${\mathrm{R}}_{\mathrm{s}}$ is the sheet resistance of TCFs, L is the length of the TCFs between the two polos, and W is the width of the TCFs. By Formula (3), we could calculate the final sheet resistance of the film. In our test, the length of the TCFs between the poles is 20 mm, and the width of the TCFs is 30 mm.

**Figure 7:**
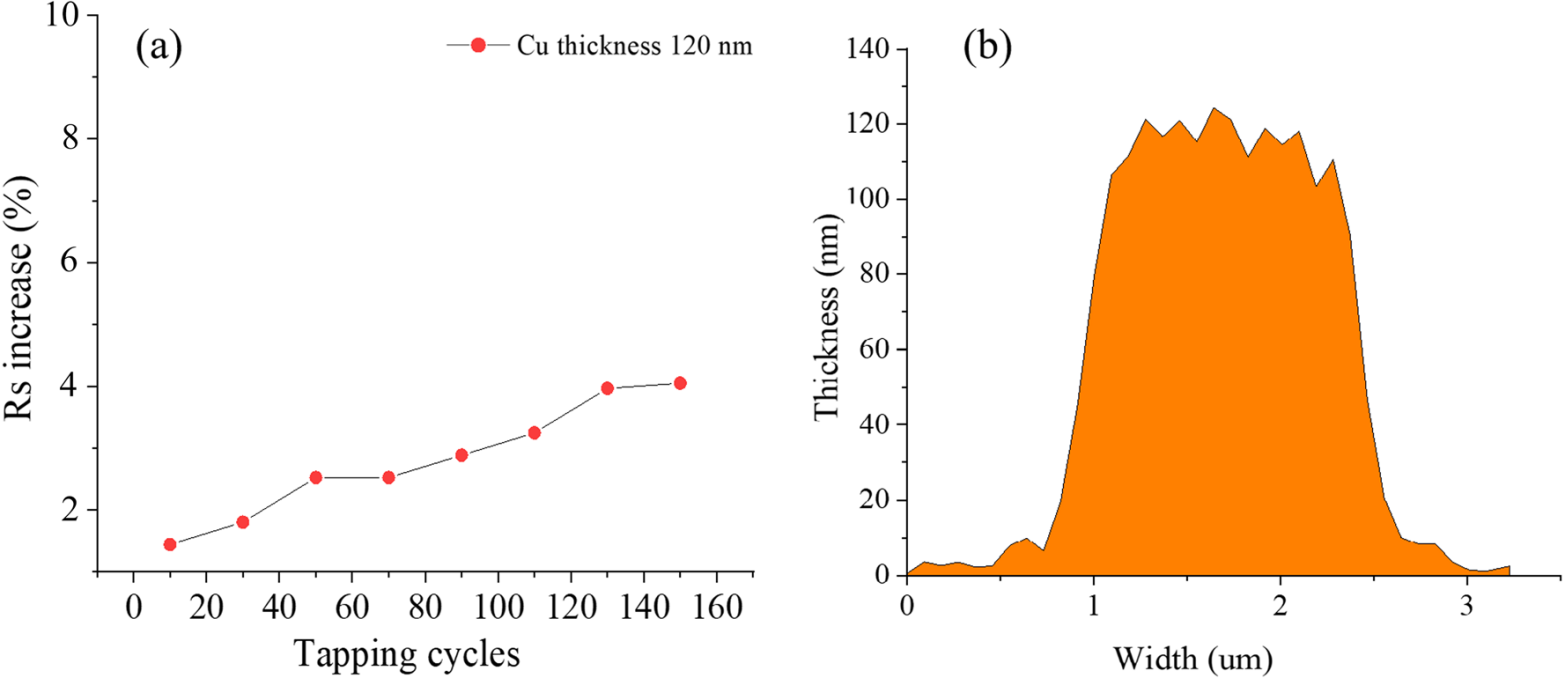
(**a**) Variation graph of sheet resistance during the adhesive tape test and. (**b**) AFM diagram of copper wire thickness.

Mechanical stability of a copper network TCF were tested by 3 M adhesive tape and cyclic bending^38^. The tapping test was conducted to evaluate the adhesion between the copper network film and PET substrates. Figure 7a shows the variation curve of square resistance with adhesive tape test times. After 160 cycles, the sheet resistance of the TCFs changes slightly, about 3%. Indicating that the copper network film has good adhesion. Figure 7b shows the thickness of the copper network wire and the thickness is about 120 nm.

As cyclic bending, we bend with the help of a common pen, which has a diameter of 9 mm as shown in the built-in diagram. We bent 600 times and measured the resistance every 100 times. The data obtained are shown in Table 1.

**Table 1:** Bending test data

Number of bending	Resistance	Sheet resistance	Rate of change of sheet resistance
0	5.1	7.65	0%
100	5.2	7.8	1.96%
200	5.3	7.95	1.92%
300	5.4	8.1	1.89%
400	5.6	8.4	3.70%
500	5.9	8.85	5.30%
600	27.2	40.8	361%

**Figure 8:**
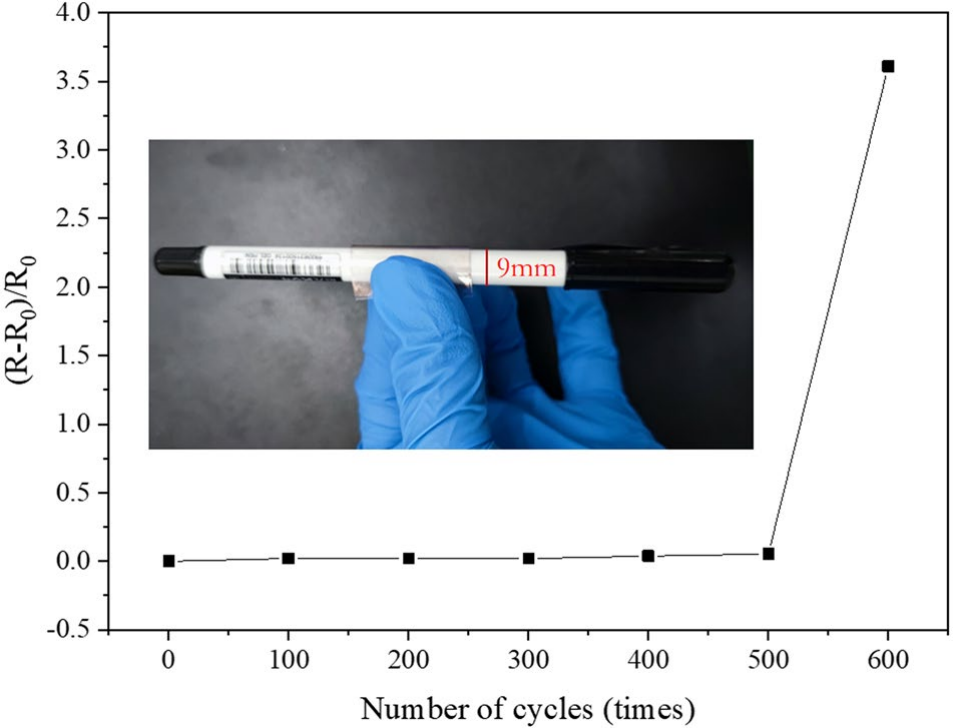
Bending test of the flexible transparent films.

**Figure 9:**
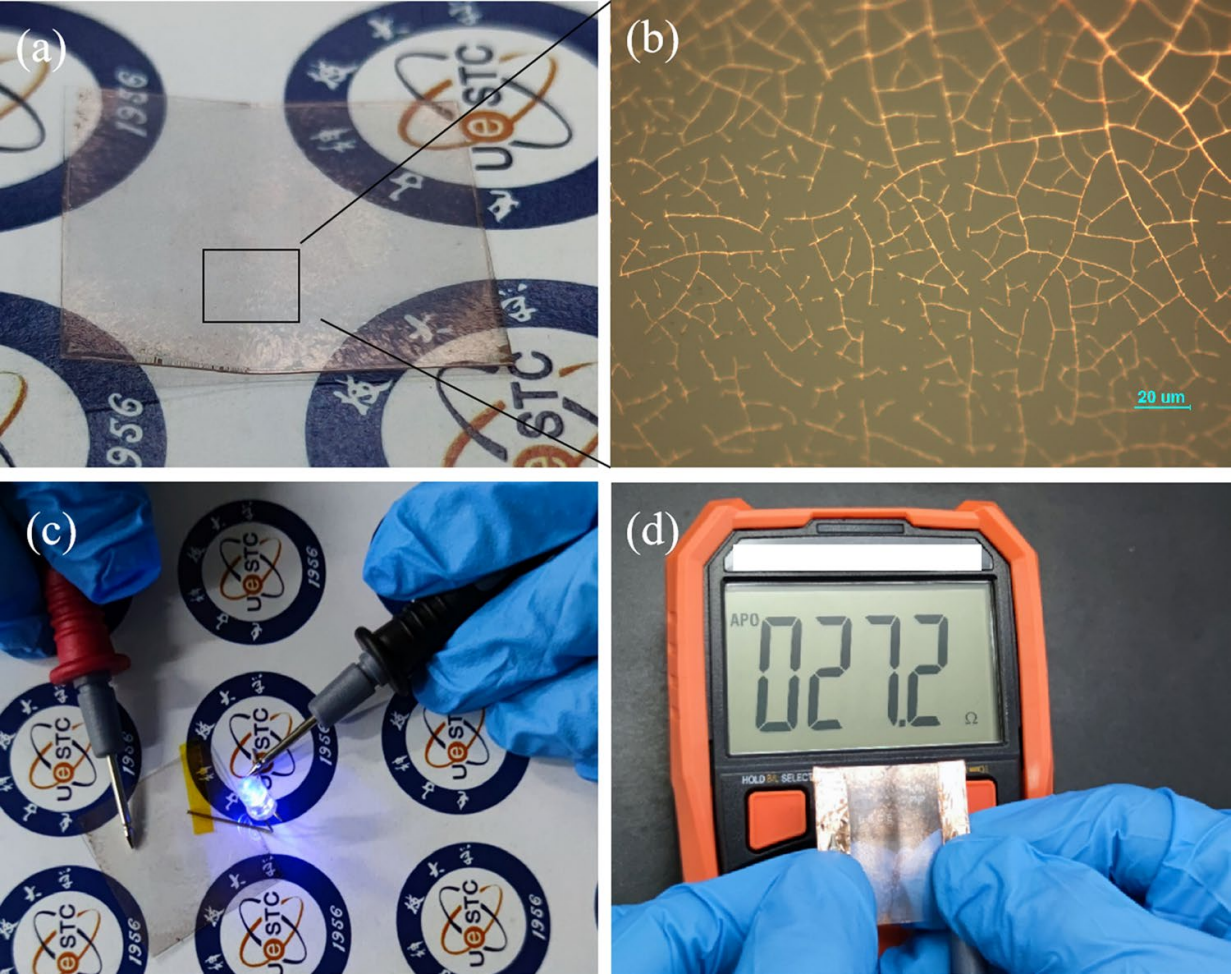
Characterization of TCFs after bending test, (**a**) TCFs after bending, (**b**) optical microscope at the crease, (**c**) schematic diagram of conductivity of TCFs, (**d**) resistance of TCFs after bending.

Figure 8 shows the result of cyclic bending test of a copper network TCF. During 500 times bends, our copper network TCF maintains low sheet resistance. When the bending times exceeds 500, the sheet resistance increases rapidly with the increase of bending times. After bending 600 times, the film shows obviously signs of bending, as shown in Fig. 9a. The network film at the crease of the TCFs was observed by optical microscope, see in Fig. 9b. There are some fractures of copper network film at the crease. However, we found that the film still has conductivity, as shown in Fig. 9c, the conductivity of the film was tested with a multimeter and a light-emitting diode. When the conductivity was measured again, it was found that the resistance of the film had changed from initial 5.1 Ω to 27.2 Ω, as shown in Fig. 9d.

### Photoelectric performance of the flexible OLED based on copper network film

**Figure 10:**
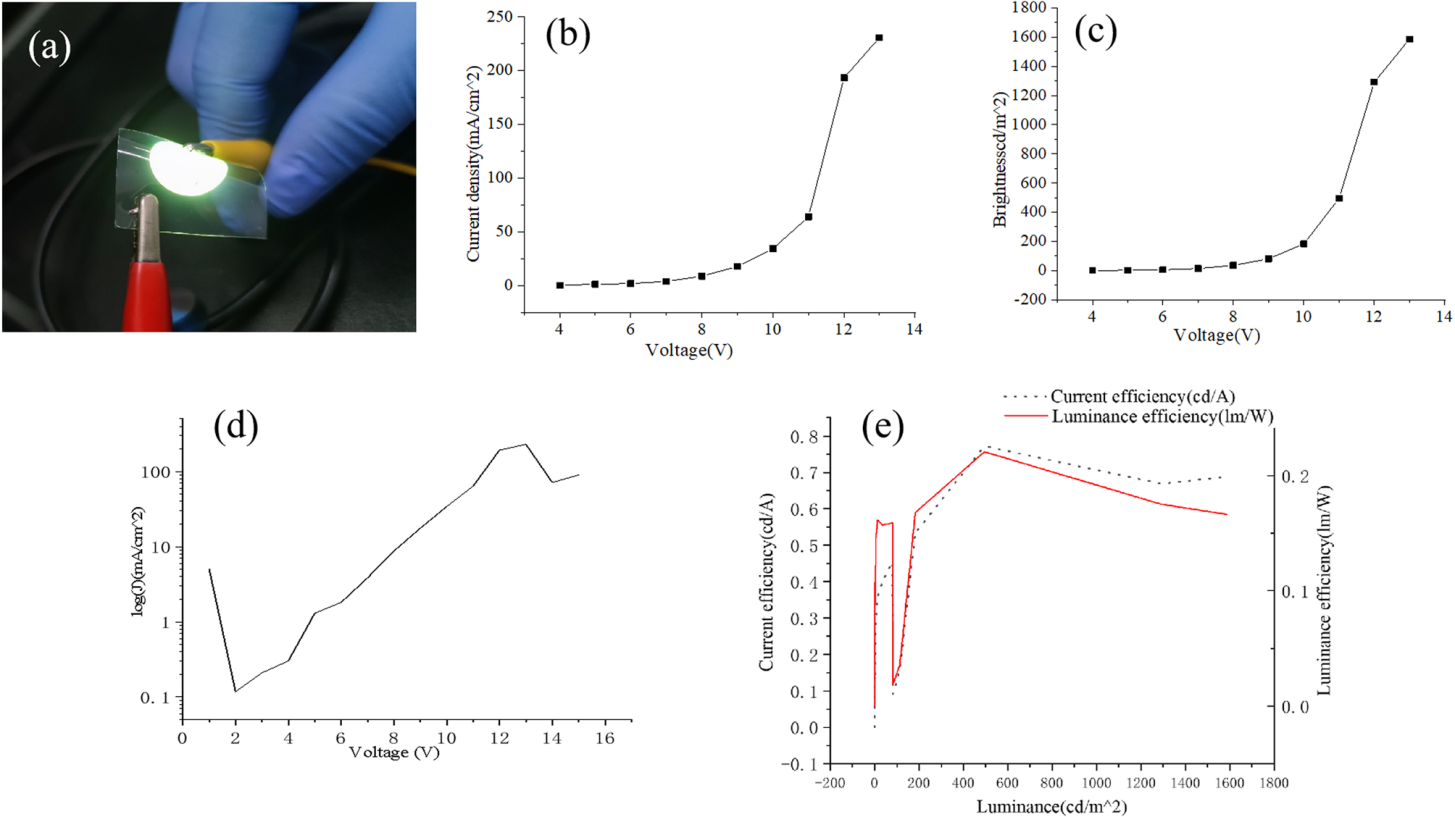
Photoelectric performance of the flexible OLED based on copper network film. (**a**) Luminescence diagram of flexible OLED at 10 V. (**b**) Current density–voltage characteristic of OLED device. (**c**) Brightness-voltage characteristic of the OLED. (**d**) Current density in log scale versus voltage. (**e**) Current efficiency and power efficiency of the OLED.

Keithley 2400 digital source meter and PR655 spectrometer system were used to test the performance of OLED. When the brightness reaches 1 cd/m^2^ driven by the voltage, this voltage is called threshold voltage. The luminance of our OLED reaches 1.679 cd/m^2^ at 5 V, that indicates the starting voltage of our device is below 5 V. Figure 10a shows a uniform light of OLED without specks, in addition, it’s also bright under curved. It’s worth noting that an excessive rate of evaporation will lead to a lack of compactness of the organic functional layer, which leads to nonuniform brightness. The evaporation rate of organic layer in this experiment is 0.2 A/s. Figure 10b, c show the current density–voltage characteristic and brightness-voltage characteristic of the OLED, respectively. The maximum luminance of the OLED was 1587 cd/m^2^. As the voltage increases, the current density increases, so does the brightness. Beyond a certain voltage, the current rise sharply. While the brightness increases rapidly along with the current density increases. However, as the voltage continues to increase, the OLED performance is declining rapidly. The possible reason for this phenomenon is that as the voltage continues to increase, the current density increases, resulting in increasing of joule heat, and then leads to an increase in temperature. The rise in temperature destroys the organic functional layer, so that it reduces the probability of effective recombination of excitons. Thus, the performance of the OLED is affected. Figure 10d is the current density in log scale versus voltage for the OLED. Figure 10e demonstrates current efficiency and power efficiency of the OLED.

## Conclusion

We demonstrated a facile fabrication strategy for copper network TCFs, based on a crack template. This method does not need complex stripping and transfer processes, what’s more, the crack template is universal for any type of metallic or substrate material. The copper network film exhibited a high transmittance of about 93% at 550 nm with sheet resistance of 13.4 Ω/sq. The figure of merit is about 380, which comparable to ITO. Another copper network TCFs produced by this method exhibited transmittance 76.5% with sheet resistance of 3.4 Ω/sq, at a copper film thickness of 120 nm. The mechanical properties network film was also investigated based on these copper network TCFs. During 500 times bends, the copper network TCFs maintain a low sheet resistance. In addition, the demonstration of flexible OLED prepared using the TCFs suggests its promising applicability. The threshold voltage of the OLED is less than 5 V and the maximum luminance is 1587 cd/m^2^. The crack template method for preparing copper grid TCFs may provide an alternative route for low-cost and large-area production of a variety of other device components, such as organic solar cells, transparent heater and supercapacitor.

## Data Availability

All data generated or analysed during this study are included in this published article.
